# Finding Furfural Hydrogenation Catalysts *via* Predictive Modelling

**DOI:** 10.1002/adsc.201000308

**Published:** 2010-09-02

**Authors:** Zea Strassberger, Maurice Mooijman, Eelco Ruijter, Albert H Alberts, Ana G Maldonado, Romano V A Orru, Gadi Rothenberg

**Affiliations:** a Van ‘t Hoff Institute of Molecular Sciences, University of AmsterdamScience Park 904, 1098XH Amsterdam, The Netherlands; b Department of Chemistry & Pharmaceutical Sciences, Vrije Universiteit AmsterdamDe Boelelaan 1083, 1081 HV Amsterdam, The Netherlands

**Keywords:** carbenes, descriptor modelling, furfural, multicomponent reactions, QSAR, quantitative structure-activity relationship

## Abstract

We combine multicomponent reactions, catalytic performance studies and predictive modelling to find transfer hydrogenation catalysts. An initial set of 18 ruthenium-carbene complexes were synthesized and screened in the transfer hydrogenation of furfural to furfurol with isopropyl alcohol complexes gave varied yields, from 62% up to >99.9%, with no obvious structure/activity correlations. Control experiments proved that the carbene ligand remains coordinated to the ruthenium centre throughout the reaction. Deuterium-labelling studies showed a secondary isotope effect (*k*_H_:*k*_D_=1.5). Further mechanistic studies showed that this transfer hydrogenation follows the so-called monohydride pathway. Using these data, we built a predictive model for 13 of the catalysts, based on 2D and 3D molecular descriptors. We tested and validated the model using the remaining five catalysts (cross-validation, *R*^2^=0.913). Then, with this model, the conversion and selectivity were predicted for four completely new ruthenium-carbene complexes. These four catalysts were then synthesized and tested. The results were within 3% of the model’s predictions, demonstrating the validity and value of predictive modelling in catalyst optimization.

## Introduction

Although it is one of the most studied processes in the history of chemistry, catalytic hydrogenation is still full of surprises.^1^ Changing the catalyst, by slightly altering a ligand or switching to a different metal precursor, often wreaks havoc in the results. Indeed, it seems that the more knowledge that is gathered on hydrogenation reactions, the more pathways and opportunities emerge for hydrogenation catalysts.^2^,^3^ Polymers, arenes and heteroaromatics are just three recent examples.^4^

**Figure 1:**
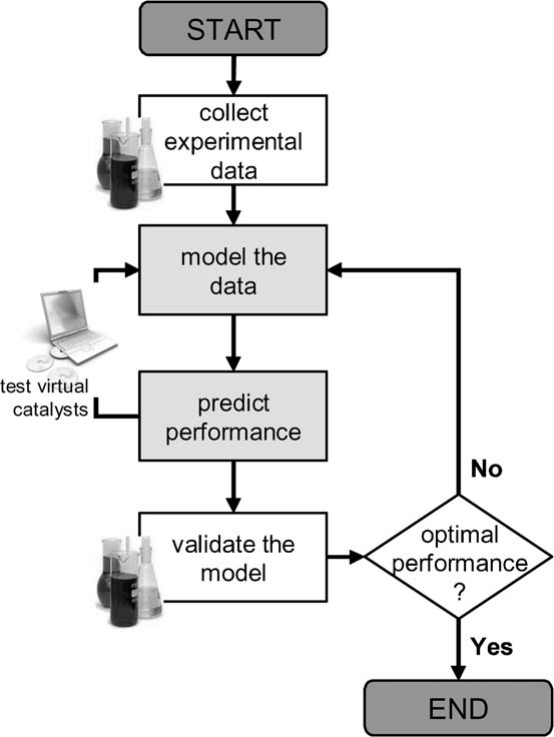
Flowchart of iterative modelling and experiments cycle.

The problem is that whilst research on homogeneous catalytic hydrogenation has provided us with effective solutions to specific reactions, there is no “grand unified theory” for finding good hydrogenation catalysts. Indeed, such a panacea is, perhaps, unrealistic. Yet, one way to move towards this goal is by teaching “catalytic intuition” to a computer,^5^ and harnessing this computational power to help solve problems in catalytic hydrogenation. As we recently showed,^6^ predictive modelling can select active regions in the catalyst space, provided that the following three conditions are met: first, you need sufficient experimental data for building a predictive model; second, you should have acces to a large number of diverse ligand-metal complexes; and, finally, a robust model validation procedure must be available. The computer can then “synthesize” sets of virtual catalysts (see flowchart in Figure 1), and predict their characteristics (descriptor values) and performance (figures of merit). These predictions can then be validated experimentally, closing the cycle.

**Scheme 1:**
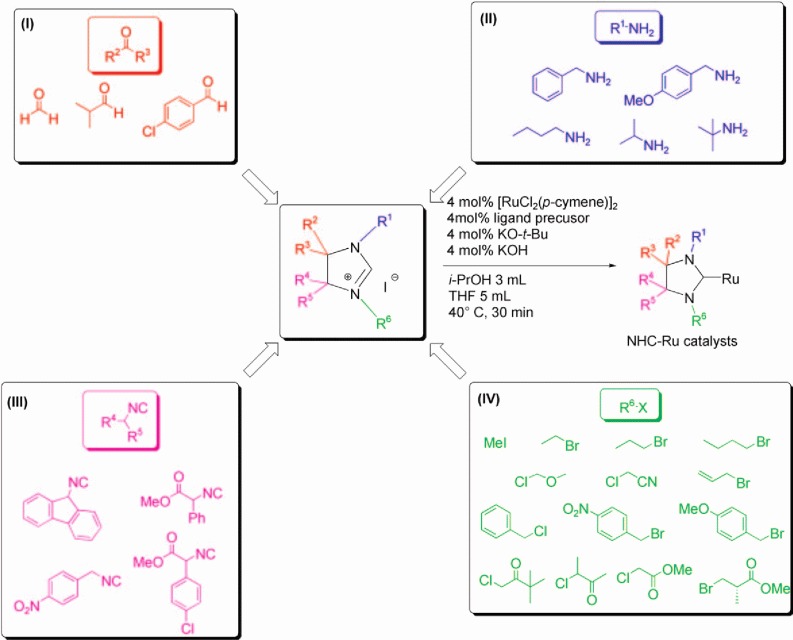
Multicomponent synthesis-alkylation-complexation route to N-heterocyclic carbenes. Using the 29 building blocks shown here, you can make 1,152 different ligands.

The most fallible step in studies of this kind is, in fact, the experimental validation. Generating large virtual libraries *in silico* is relatively easy, but restricting these according to explicit synthetic rules often robs them of their diversity. To avoid this pitfall, we turned to making our catalysts *via* multicomponent synthesis protocols. Multicomponent reactions (MCRs) are convergent procedures that combine at least three simple, easily accessible building blocks in a one-pot process, thus powerful reactions for generating functionalized complex molecules.^7^–^15^ MCRs proceed with remarkably high atom and step economy by reducing the number of functional group manipulations, thus avoiding the use of protective groups and, as such, are ideally suited for the generation and validation of catalyst libraries. Previously, we have presented the scope and application of MCRs for synthesizing 2*H*-2-imidazolines^14^,^15^ which are key intermediates *en route* to N-heterocyclic carbene (NHC) complexes,^14^ some of today’s most promising hydrogenation catalysts.^16^ In this MCR (Scheme 1), an aldehyde or ketone **I**, a primary amine **II** and an α-acidic isocyanide **III** are combined in a one-pot reaction, giving the corresponding 2*H*-2-imidazoline. The product is then easily alkylated with an alkyl halide **IV** at position N-3, yielding the final NHC precursor.

In this paper, we demonstrate the utility of combining multicomponent ligand synthesis and catalytic performance studies with predictive modelling for catalyst discovery and optimization. Our case study focuses on catalysts for hydrogenating furfural **1** to furfurol **2** [Eq. (1)]. This is an industrially important reaction,


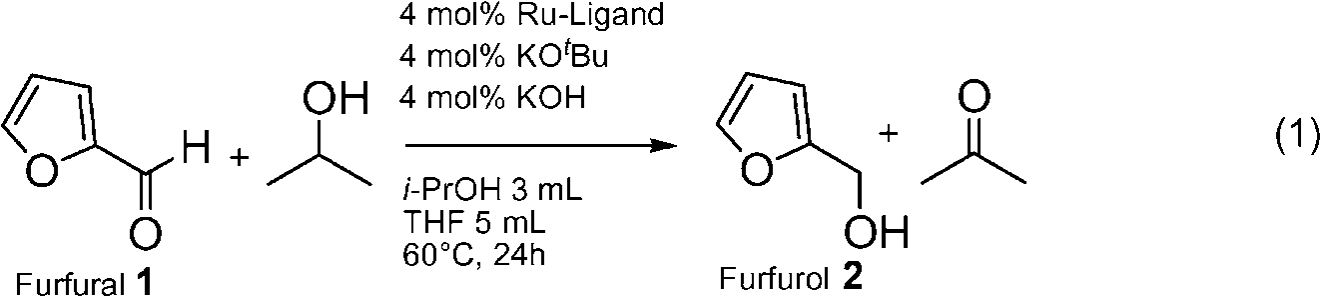
(1)

as furfural is readily obtained from cellulosic biomass,^17^,^18^ and its hydrogenation is a key pathway in bio-refining.^19^ Moreover, the presence of both a heteroaromatic ring and a carbonyl group makes furfural a versatile study subject, from which one can draw analogies to many similar compounds. Recently, we showed the first proof of concept for using this MCR in the synthesis of transfer hydrogenation catalysts.^20^

## Results and Discussion

The multicomponent synthesis gives us access to a broad range of imidazolinium precursors. In fact, the range is already too broad for traditional synthesis and screening studies. Using just the 29 building blocks from Scheme 1 (all of which are commercially available) already gives 1,152 different ligand precursors. In theory, we could synthesize and test all of them. In practice, having a model that can pinpoint the active regions in this catalyst space is much more efficient and convenient. We want to minimize the number of experiments, without compromising the search for good catalysts. This is where the iterative cycle of experiments, descriptor modelling, and validation comes in.

**Scheme 2:**
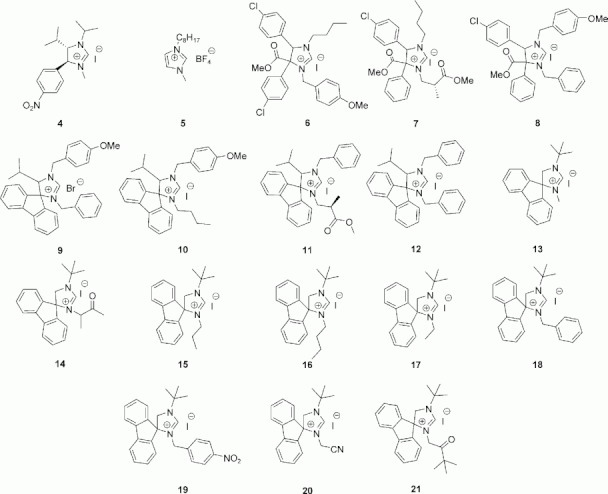
Chemical structures for the 18 ligands synthesized in the first iteration.

We prepared a set of 18 imidazolinium salts following the synthetic pathway shown in Scheme 1 (see Experimental Section for details). These ligand precursors (structures **4**–**21** in Scheme 2) were isolated and purified prior to the catalysis screening tests. Then, in a typical reaction [Eq. (1) above], an equivalent amount of the imidazolinium precursor was deprotonated with potassium *tert*-butoxide for 30 min at 40 °C, and the resulting carbene was coordinated *in situ* to the dimer Ru salt precursor, [RuCl_2_(*p*-cymene)]_2_. Formation of this complex was shown clearly by ^13^C NMR.^21^ Both reactions were complete within 30 min. Subsequently, an excess of isopropyl alcohol, used as both solvent and sacrificial hydrogen donor, was added, together with one equivalent of potassium hydroxide, activating the catalyst. Then, twenty-five equivalents of furfural were added and the mixture was heated to 60 °C and stirred under nitrogen for 24 h. Reaction progress was monitored by GC and ^1^H NMR (detailed procedures are given in the Experimental Section and the Supporting Information).

Table 1 shows the substrate conversion, product selectivity and product yield. In the absence of any carbene ligand, the metal precursor [RuCl_2_(*p*-cymene)]_2_ already gives 94% conversion, with 84% yield of furfurol after 24 h. Nevertheless, adding a carbene ligand can raise both numbers to >99.9%. Control experiments showed that the hydrogen transfer reaction is sensitive to the type and amount of base used. The best conversions were obtained with a mix of potassium *tert*-butoxide for deprotonating the imidazolinium precursor, and KOH as promoter.^22^ No reaction occurred in the absence of KOH, and <80% yield was observed when using 0.1 equivalents of KOH with respect to the Ru catalyst.

**Table 1:** Catalytic transfer hydrogenation of furfural 1 to furfurol 2.^[a]^

Entry	Imidazolinium Ligand Precursor	Furfural	Furfurol
		Conversion [%][a]	Selectivity [%]	Yield [%][b]
1	**4**	100.0	99.9	99.9
2	**5**	95.9	99.1	95.0
3	**6**	97.9	99.4	97.4
4	**7**	62.4	98.9	61.7
5	**8**	89.1	98.8	88.0
6	**9**	100.0	98.3	98.3
7	**10**	99.9	99.5	99.4
8	**11**	63.3	99.2	62.8
9	**12**	98.9	98.1	97.1
10	**13**	98.9	98.1	97.0
11	**14**	99.8	98.3	98.2
12	**15**	99.4	98.7	98.2
13	**16**	98.8	99.2	98.1
14	**17**	99.2	98.5	97.8
15	**18**	99.3	99.6	99.0
16	**19**	99.9	99.6	99.5
17	**20**	100.0	99.3	99.3
18	**21**	91.6	98.2	90.0
19	no ligand	94.1	90.0	84.6

[a] *Standard reaction conditions:* 5 mmol furfural **1**, 0.1 mmol [RuCl_2_(*p*-cymene)]_2_; 0.2 mmol 2-imidazolinium precursor (structures shown in Scheme 1); 0.2 mmol KO*-t-*Bu; 0.1 mmol KOH; 5 mL dried THF; 3 mL *i-*PrOH; N_2_ atmosphere; 60 °C; 24 h and with a minimal TON of 20–25 (4% catalyst).

[b] Yield determined by ^1^H NMR analysis as well as GC analysis (*n*-octane as internal standard).

One question that immediately springs to mind in such systems pertains to the ligand dissociation equilibria: what is the chance that, in the course of the catalytic cycle, the carbene ligand(s) dissociate from the Ru complex? However, our carbene ligands remain coordinated throughout the cycle, since any free carbene in solution would lead to immediate acyloin condensation, analogous to the reactions reported by Enders et al.^23^ Control experiments showed that the acyloin condensation [Eq. (2), giving roughly 90%


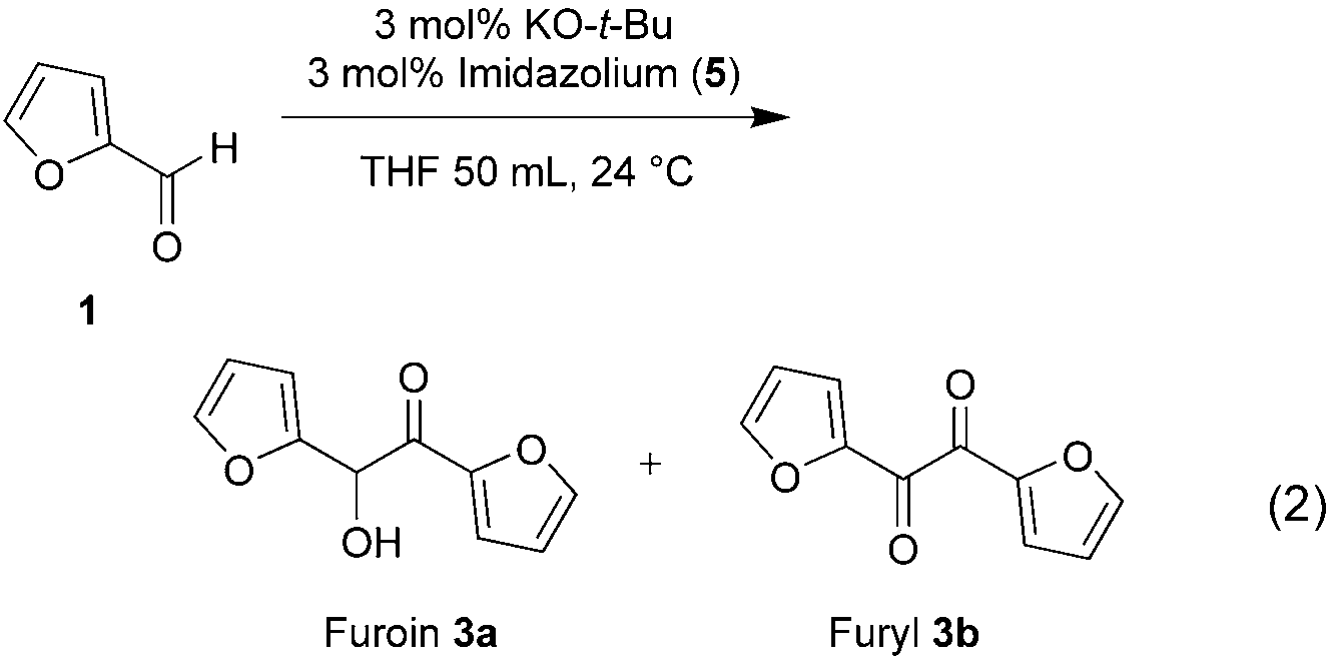
(2)

furoin **3a** and 10% furyl **3b**] is indeed very fast compared to the transfer hydrogenation reaction, with *t*_1/2_ <15 min.^24^ Thus, by using a 1:1 ligand:Ru ratio, we managed to avoid any acyloin condensation, confirming that the carbene ligand remains coordinated to the ruthenium complex throughout the catalytic cycle.

**Figure 2:**
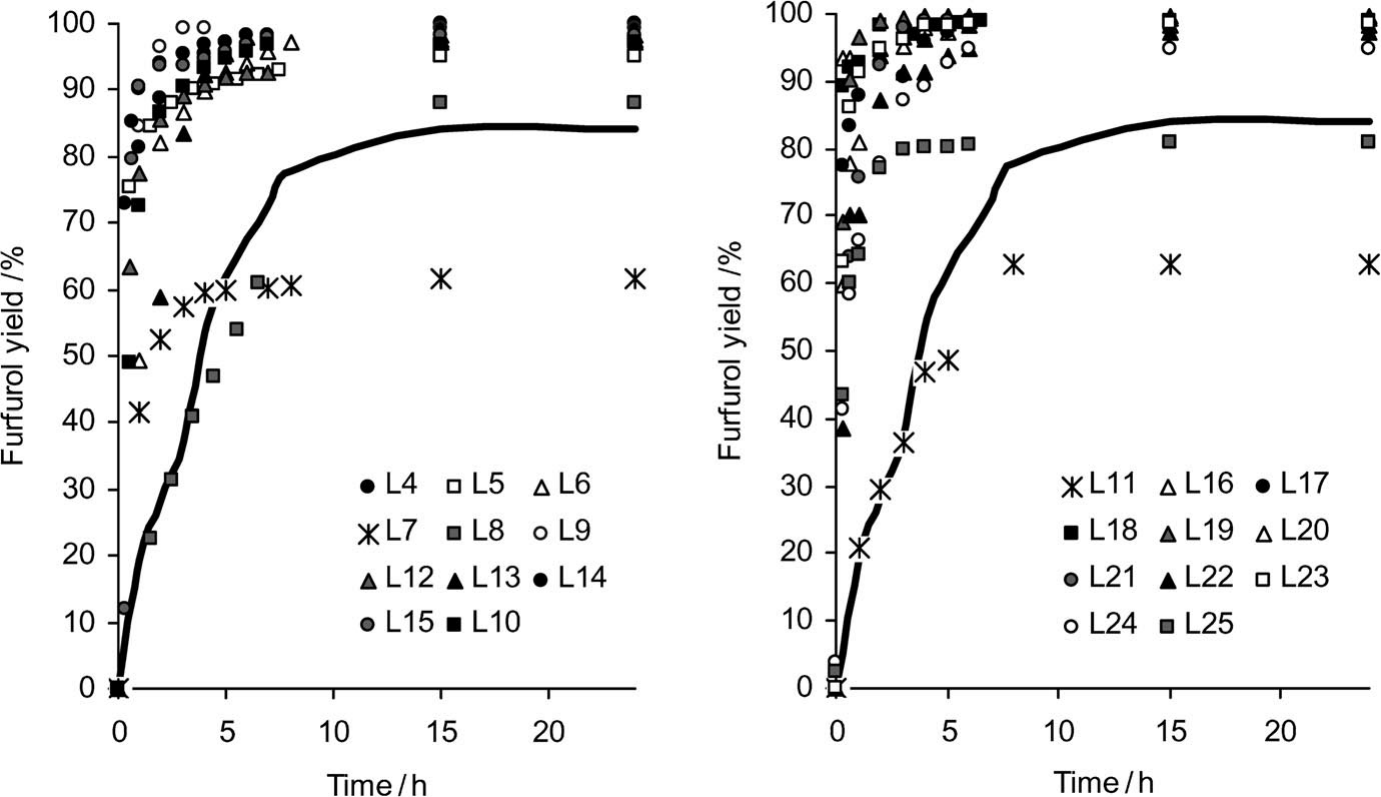
Time-resolved reaction profiles for the Ru-carbene catalyzed hydrogenation of furfural 1 to furfurol 2, following the standard reaction conditions as shown in Table 1. For clarity, the data are divided in two groups (*left* and *right* graphs), since several profiles overlap. The blank experiment using [RuCl_2_(*p*-cymene)]_2_ precursor only (no ligand) is shown as a thick continuous curve.

Figure 2 shows time-resolved reaction profiles for the transfer hydrogenation reaction catalyzed by the 18 different ruthenium-carbene complexes made from the imidazolinium salts in Scheme 2, where L**n** denotes the ligand prepared using precursor **n**. For completeness, this figure also shows the profiles obtained with the catalysts synthesized later, following the model predictions (L**22**–L**25**) The catalysts are divided in two groups (*left* and *right* graphs) for clarity. The yield obtained without carbene ligand (i.e., just using the [RuCl_2_(*p*-cymene)]_2_ precursor) is shown as a continuous curve. We see that several of the Ru complexes, and especially those derived from imidazolinium salts **4**, **9** and **19**, exhibit both fast and highly selective catalysis.

**Scheme 3:**
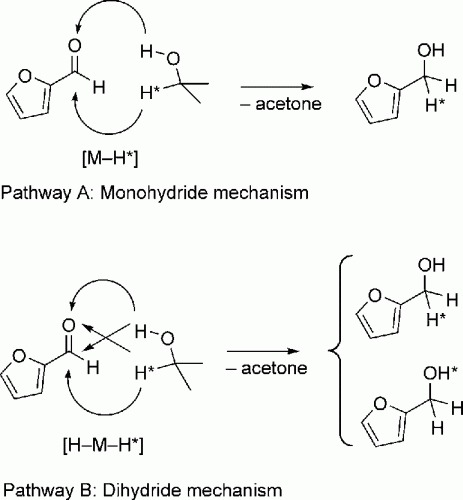
Two possible transfer hydrogenation pathways from *i-*PrOH to furfural.

Delving deeper into the reaction mechanism, we tried to determine a pathway for the transfer hydrogenation process.^25^ Ruthenium-catalyzed hydrogen transfer typically involves either a monohydride or a dihydride species in the catalytic cycle. The major difference lies in the location of the hydride in the hydrogenated product, as shown by the elegant isotope substitution experiments of Bäckvall’s group^26^ and Enthaler and co-workers.^22^ If the catalytic cycle follows the monohydride pathway (Scheme 3, *top*), the hydrogen bound to the carbinol carbon donor is transferred only to the carbonyl carbon. Conversely, in the dihydride pathway, the C—H and O—H from the hydrogen donor are equivalent with respect to the hydrogen transfer. Since hydroxide protons exchange rapidly with solvent protons, and since the transfer hydrogenation is reversible, the incorporation of labelled hydrogen in the product OH group will not exceed 50% (Scheme 3, *bottom*). In our case, a series of experiments using isotopically labelled (CH_3_)_2_CD(OH) gave a rate constant ratio of *k*_H_:*k*_D_=1.5. This value implies a secondary deuterium isotope effect, corresponding to a route where the scission of the isopropyl alcohol C—H bond is not the rate-determining step. Moreover, following the reactions with ^1^H NMR showed an 80:20 incorporation ratio of deuterium on the carbon and the oxygen, respectively. This implies at least partial participation of the monohydride pathway.^27^

These experimental results are ideal for modelling purposes, since the data show a wide range and spread of activity values.^28^,^29^ Interestingly, pairing the reaction profiles in Figure 2 with the imidazolinium salt structures in Scheme 2, one sees two things: first, the type of NHC ligand influences the catalytic activity. In most cases, the ligand enhances the catalysis, but not always. Second, we cannot point to any common structural factor that influences catalytic activity. For example, one would expect complexes derived from the precursor pairs {6, 7} and {11, 14} to give similar performance, due to their respectively similar backbone structure,^6^ but the results show otherwise. In each of these pairs, one ligand is an extremely good catalyst while the other is a poor catalyst. In the case of 7, the performance is even lower than that of the carbene-free blank run! The elusiveness of a simple explanation that tallies with our ‘chemical intuition’ does not mean that it does not exist. It simply means that simple ChemDraw structures as depicted in Scheme 2 give us insufficient information. Moreover, the larger and more diverse the input data set is, the more difficult it becomes to predict activity based on chemical intuition alone.

To solve this problem, we applied predicting modelling on our dataset. The idea behind building a predictive model is linking the ligand-complexes space (the imidazolinium precursors) with a figure of merit or measured response (e.g., the furfural hydrogenation yield), through an intermediate space of molecular descriptors. A descriptor is a number that encodes structural and/or chemical information.^30^,^31^ The ligand backbone, the cone angle and the reaction pocket volume are well known examples. By choosing the “correct” descriptors we can build a model that takes them as input and gives the predicted values of performance (furfurol yield) as output. However, choosing the right descriptors for modelling a given catalytic system is both crucial and difficult. Previously, we showed that one can select good descriptors by simply calculating a large set of them and then sieving out the less important ones by using, for example, variable importance (VIP) analysis.^32^,^33^ Here, we combined two statistical methods, principal component analysis (PCA) and partial least squares (PLS) regression, to build and validate our descriptor model (see Experimental Section for details).

**Figure 3:**
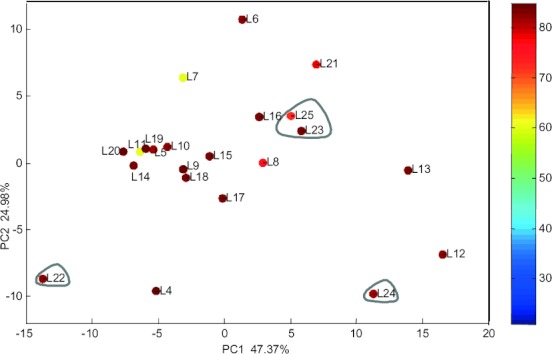
Scores plot for the Ru complexes of the 2-imidazolinium salts used in this study.

First, we calculated a large number of various descriptors for all of the catalysts derived from the precursors shown in Scheme 2 (168 descriptors for each catalyst, see summary in Table 2 and complete list in the Supporting Information). Then, we ranked these descriptors according to their importance and examined the correlation between them using PCA. Figure 3 shows the relative position of the catalysts based on the two first PCs (the so-called ‘scores plot’). These explain >72% of the variance in the data. We see that the catalysts are well distributed. This is important since experiments that are “bunched together” in a scores plot are redundant. Thus, Figure 3 shows that this dataset is sufficiently diverse for building good predictive models. There is a single cluster, composed of ligands **5**, **10**, **11**, **14**, **19** and **20**. No outliers are observed, but low-yield data are scarce. This means that a model based on these data will be biased towards high yields. For modelling purposes, low-yield experiments are just as important as high-yield ones.^6^

**Table 2:** Definition and labelling of the descriptor types shown in Figure 4.

Label	Type	Examples
○	2D, constitutional	Elemental composition, atom counts, atom types (no geometric, no electronic information)
×	2D, topological	Connectivity, paths, shape and flexibility of the molecule
>	3D, geometrical	Inertia moment, 3D shadows, volume, surface, gravitation index
+	3D, electrostatic	Charge distribution, electro negativity, polarity

**Figure 4:**
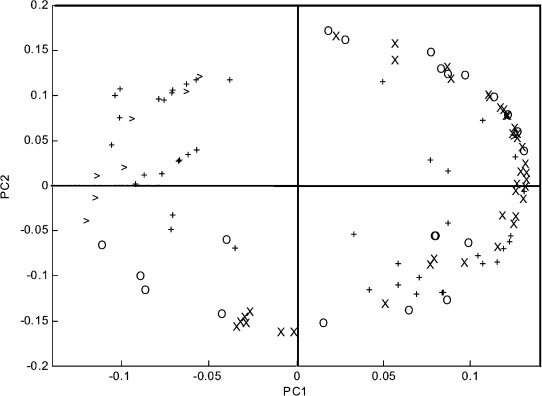
Loadings plot for the 168 descriptors. In order to study the effect and the relevance of the descriptor families in the PC space, we have added different labels to different descriptors. See Table 2.

The loadings plot, shown in Figure 4, indicates how much each descriptor contributes to a given PC. Unimportant descriptors have small loading values, and will appear close to the centre. Conversely, important descriptors will appear far from the centre. In this plot there are no symbols close to the centre. This means that all the variables are roughly equally important. The 2D constitutional descriptors (‘○’ symbols), appear in the top right and bottom left quarters, contributing equally to both PC1 and PC2. Similarly, the 3D electrostatic descriptors (‘+’ symbols) contribute equally to the first two PCs. Thus, these types are the more important descriptors. Conversely, the 3D geometrical descriptors (‘>’ symbols) appear mainly on the right half of the plot, and the 2D topological descriptors (‘×’ symbols) appear mainly on the top left quadrant, so these are less relevant for PC1 and PC2, respectively.

**Figure 5:**
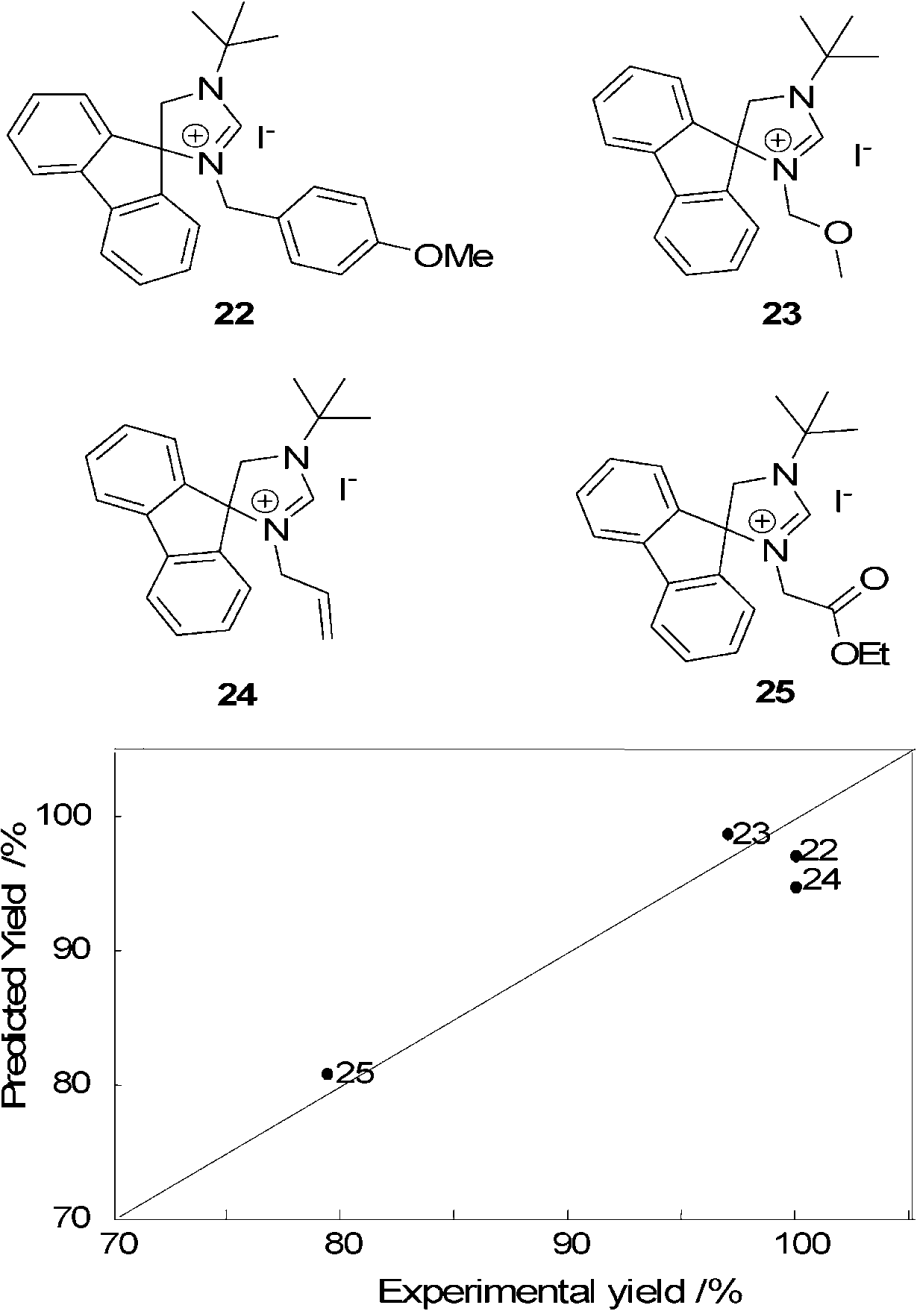
The four new ligands 22–25 (top) and their predicted *vs.* experimental yield (bottom).

To predict the catalytic performance of new carbene-ruthenium complexes in this reaction, we used a PLS regression model. We divided the original 18 ligand precursors (structures **4**–**21**) in a training set of 13 and a validation set of five ligand precursors, which resulted in a promising *R*^2^=0.913.^34^ Then, the predictive power of the model for actual new structures was evaluated. As noted above, the multicomponent reaction provides facile access to a large number of possible ligands. We predicted the activity and selectivity of various structures using the model, and chose to synthesize four new ligand precursors (structures **22**–**25** in Figure 5). Figure 5 and Table 3 show the results. The experimental tests show that our average prediction error is ∼3%. This is quite small considering the near-quantitative yield. With the large number of data points in the high-yield area, however, such a high variation is somewhat surprising. It may reflect a similarity in the test set structures. Importantly, the model was not only able to find high-yield compounds, but also a low-yielding one (ligand **25**). These results demonstrate the value of using predictive descriptor modelling, since drawing these four ligands and calculating their descriptors is much less work than their actual synthesis.

**Table 3:** Predicted *vs.* experimental yield for ligands 22–25.

Ligand	Predicted yield [%]	Experimental yield [%]	Error [%]
**22**	100.0	97.2	2.9
**23**	96.9	98.7	1.8
**24**	100.0	94.8	5.4
**25**	79.4	80.8	1.7

## Conclusions

Combining multicomponent synthesis and predictive modelling facilitates the quest for new catalysts. MCRs give easy access to a variety of potential ligands, each of which can be synthesized in high yields and selectivities. Predictive modelling guides the synthesis efforts by highlighting “good regions” in the catalyst space. In this way, one can avoid “dead ends” and focus the experimental effort on promising structures. Importantly, we have shown that descriptor models can give high correlations even in situations where structure/activity relationships are elusive. Moreover, this combined approach allows facile simultaneous variation of the parameters (e.g., changing substituents on several sites of the heterocyclic ring) since in the descriptor model the dataset is taken as a whole. We believe that such an approach will expand rapidly, enhancing the performance of conventional catalysis research. That said, computers will not replace chemists, and data mining methods will not replace mechanistic studies. These methods will simply be part of the chemist’s toolbox in the 21^st^ century.

## Experimental Section

### Materials and Instrumentation

GC analysis was performed using an Interscience GC-8000 gas chromatograph with a 100% dimethylpolysiloxane capillary column (VB-1, 30 m×0.325 mm). ^1^H and ^13^C nuclear magnetic resonance (NMR) spectra were recorded on Varian Mercury *300* (300 MHz for ^1^H), Bruker Avance 250 (250.13 MHz for ^1^H and 62.90 MHz for ^13^C), or Bruker Avance 500 (500.23 MHz for ^1^H and 125.70 MHz for ^13^C). Chemical shifts (*δ*) reported in reported in ppm, internally referenced to residual solvent resonances for CDCl_3_ (^1^H δ=7.26 ppm; ^13^C{^1^H} δ=77.00 ppm), and coupling constants (*J*) are reported in Hz. Samples for GC analysis were diluted in 1 mL EtOH. GC conditions: isotherm at 60 °C (2 min); ramp at 50 °C min^−1^ to 80 °C; isotherm at 80 °C (3 min); ramp at 1 °C min^−1^ to 90 °C; ramp at 50 °C min^−1^ to 250 °C; isotherm at 250 °C (3 min). All reactions were performed under N_2_ using standard Schlenk techniques. Unless otherwise noted, all chemicals were purchased from commercial sources and used as received. All products are known compounds and were identified by comparing of their GC retention times and/or NMR spectra to those of authentic samples. Ligands (**4**, **9**, and **13**) are known compounds, and were synthesized following a published procedure^14^,^15^ Ligand **5** was purchased from Fluka. The other eighteen ligands are new compounds, synthesized using a modified procedure as outlined below. Detailed synthesis procedures and characterization data for these compounds, as well as details of the NMR characterization control experiments, are included in the Supporting Information.

### General Procedure for the Synthesis of 2*H*-2-Imidazoline Archetypes

Seven archetypes of 2*H*-2-imidazolines (**A**–**G**), the structures of which can be found in the Supporting Information, were synthesized *via* a three-component Mannich-type reaction according to literature procedures ([**A**–**D**]: ref.^16^, [**E** and **F**]: ref.^9^, **G**: ref.^12^).

### General Procedure for the Synthesis of 2-Imidazolinium Salts

Reactions were carried out at a concentration of 1 M of imidazoline in acetone. The halide (1 equiv.) was added to a stirred solution of the 2*H*-2-imidazoline and NaI (1 equiv.). The reaction mixture was stirred at 25 °C for 18 h. Then, the reaction mixture was filtered over Celite and concentrated under vacuum.

### *Example*: Imidazolinium iodide 23


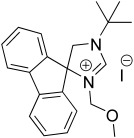
(3)

According to general procedure II for the synthesis of 2-imidazolinium salts, the reaction between 2-imidazoline **D** (297.2 mg, 1.07 mmol), NaI (160.5 mg, 1.07 mmol) and chloromethyl methyl ether (86.1 mg, 81.3 μL, 1.07 mmol) afforded 2-imidazolinium iodide **23** as a yellow foam; yield: 481.7 mg (1.07 mmol, 100%). ^1^H NMR (500 MHz, CDCl_3_): *δ*=9.87 (s, 1 H), 7.72–7.69 (m, 4 H), 7.51–7.49 (m, 2 H), 7.45–7.43 (m, 2 H), 4.757 (s, 2 H), 4.30 (s, 2 H), 3.06 (s, 3 H), 1.70 (s, 9 H); ^13^C NMR (125 MHz, CDCl_3_): *δ*=157.1 (CH), 142.4 (C), 140.1 (C), 131.1 (CH), 129.4 (CH), 124.5 (CH), 120.8 (CH), 73.6 (C), 58.7 (CH_2_), 57.8 (CH_2_), 56.9 (CH_3_), 28.1 (3 CH_3_); IR (neat): ν=2988, 2947, 2877, 1616, 1449, 1374, 1250, 1172, 1107, 1105, 918, 766, 733 cm^−1^; HR-MS: *m/z*=321.1947, calcd. for C_21_H_25_N_2_O (M−I^−^): 321.1961.

### Procedure for the Preparation of Ru-Carbene Complexes

A solution of [RuCl_2_(*p*-cymene)]_2_ (61 mg, 0.1 mmol) was stirred under N_2_ in a 25-mL Schlenk tube together with the 2-imidazolinium salt (0.2 mmol), KO-*t-*Bu (22 mg, 0.2 mmol) and KOH (6 mg, 0.1 mmol) in 5.0 mL dried THF and 3.0 mL *i-*PrOH for 30 min at 40 °C.

### *Example*: [RuCl_2_(*p*-cymene)]_2_/Ligand 4

[RuCl_2_(*p*-cymene)]_2_ (61 mg, 0.1 mmol), 2-imidazolinium salt **4** (83.4 mg, 0.2 mmol), KO*t*Bu (22 mg, 0.2 mmol) and KOH (6 mg, 0.1 mmol) were stirred in 5.0 mL dried THF and 3.0 mL *i-*PrOH for 30 min at 40 °C.

### Procedure for Catalytic Transfer Hydrogenation

In a 25-mL Schlenk tube under N_2_, the catalyst was first prepared according to the previous procedure. After adding furfural **1** (480 mg, 5 mmol) and the internal standard octane, in a furfural-octane mass ratio of 2:1, the reaction mixture was heated to 60 °C and stirred for 24 h. Periodically, samples were taken out and analyzed by GC.

### Procedure for Acyloin Condensation of Furfural 1 to Furoin 3a^23^

In a 100-mL Schlenk tube equipment under N_2_, 2-imidazolium salt (**5**) (28 mg, 1 mmol) and KO-*t-*Bu (122 mg, 1 mmol) were dissolved in 50 mL of dry THF and stirred for 15 min at 24 °C. After addition of distilled furfural **1** (3200 mg, 33 mmol) the reaction mixture was stirred overnight, and quenched with 2 mL formic acid (98%). The solvents were evaporated and the crude product was recrystallized from EtOH (85 mL), to give **3a** as a yellow solid (yield: 3030 mg, 31.56 mmol, 94%) containing 2–3% of furyl **3b** as side product.

### Procedure for Transfer Hydrogenation of Furfural with *i-*PrOH-2*d_1_* as Hydride Source

In a 10-mL Schlenk tube under nitrogen atmosphere, 0.05 mmol [RuCl_2_(*p*-cymene)]_2_, 0.1 mmol 2-imidazolinium salt **5**, 0.1 mmol KO-*t-*Bu, 0.05 mmol KOH were dissolved in 3 mL dried THF and 1.5 mL *i-*PrOH-2*d*_1_ and stirred for 30 min at 24 °C. The reaction mixture was heated to 60 °C for 5 h after the addition of 2.5 mmol furfural **1** and the internal standard, octane. The solvent was removed in vacuum and the residue solved in CDCl_3_. The conversion was determined by ^1^H NMR and by GC analysis. ^1^H NMR (300 MHz, CDCl_3_): *δ*=7.41 (s, 1 H), 6.35 (d, 1 H), 6.30 (d, 1 H), 4.70 (s,1 H), 4.62 (s, 2 H), 3.73 (s, 1 H).

### Computational Methods

All computations were performed on a Dell Latitude D630 laptop with a double core 4.0 GHz processor.

### Ligand Geometry Optimization and Descriptor Calculation

Geometry optimization for calculating the 3D descriptors was performed using Hyperchem.^35^ We used the MM+ force field in combination with a conjugate gradient optimization method (Polak–Ribiere). The 3D optimization of the ligand-metal complexes turned out to be difficult. The common algorithms for geometric optimization yielded only high energies (∼120 kcal) instead of the minima of 20–30 kcal. Having a 90° angle between the imidazole aromatic ring and the substituent aromatic 3-ring spiro moiety is crucial. The solution is to force a change into the 3D configuration. This gives a better initial structure for the geometry optimization. The descriptors were computed with the Codessa software package^36^ and analyzed using Matlab scripts.^37^ A total of 168 descriptors (2D and 3D) were calculated for four different classes: constitutional, topological, geometrical and electrostatic. A full list of these descriptors is included in the Supporting Information.

### Data Analysis and Model Validation

The descriptors matrix was analyzed using principle component analysis (PCA). A detailed description of this technique is available elsewhere.^34^,^38^ Putting it simply, PCA reduces a large data matrix into two smaller matrices, that are easier to examine and interpret. Using PCA, you can extract the key factors. These are the *principal components*, or PCs (also called the *latent variables*). Mathematically speaking, if **X** is an (*I*×*J*) matrix that contains *J* variables for *I* reactions, PCA divides this matrix into a systematic part **TP**^T^ (the PCA model), and a residuals part **E. T** is the **scores matrix**. It represents the spread of the reactions within the model space. **P** is the **loadings matrix**. It describes the relationships between the variables.

After calculating the ligand descriptors and ranking them, we used partial least squares (PLS) regression to relate them to the yield of furfurol (the figure of merit, FOM). For this model, we computed 12 latent variables, retaining the five most important ones that explained 89.2% of the variance in the data.

The model was validated using both internal and external prediction. For the internal validation, we used a training set of 13 ligands and a validation set of five ligands. Cross-validation was used to improve the model’s stability. For the external validation, we used four newly synthesized ligands, that were “unknown” to the model. The error percentage and the ΔFOM for the external validation were calculated using %error=|ΔFOM/expFOM|×100, and ΔFOM= |expFOM−predFOM|, respectively. A more detailed note on the prediction error and a residuals plot are included in the Supporting Information.

## Supporting Information

Detailed facts of importance to specialist readers are published as ”Supporting Information”. Such documents are peer-reviewed, but not copy-edited or typeset. They are made available as submitted by the authors.
