# Supplementary material for: Finding Furfural Hydrogenation Catalysts via Predictive Modelling
Source: Adv Synth Catal. 2010 Sep 2;352(13):2201–10. doi: 10.1002/adsc.201000308 (PMC3501696; doi:10.1002/adsc.201000308)

Advanced Synthesis & Catalysis

Supporting Information

© Copyright Wiley-VCH Verlag GmbH & Co. KGaA, 69451 Weinheim, 2010

Supporting information for the article

Finding furfural hydrogenation catalysts via predictive modelling

Zea Strassberger[a], Maurice Mooijman[b], Eelco Ruijter[b], Albert H. Alberts[a], Ana G. Maldonado[a], Romano V.A. Orru[b*] and Gadi Rothenberg[a*]

[a] Van 't Hoff Institute of Molecular Sciences, University of Amsterdam, Science Park 904, 1098XH Amsterdam, The Netherlands

Tel. +31 (0)20 525 6963, Fax. +31 (0)20 525 5604, e-mail: g.rothenberg@uva.nl

[b] Department of Chemistry & Pharmaceutical Sciences, Vrije Universiteit Amsterdam, De Boelelaan 1083, 1081 HV Amsterdam, The Netherlands

Table of Contents

Ligand characterization: **S2**

Materials and Instrumentation *S2*

General procedure I for the synthesis of 2H-2-imidazoline archetypes. *S2*

General procedure II for the synthesis of 2-imidazolinium salts. *S3*

Procedures for performing control NMR experiments *S9*

GC calibration experiments for furfural and furfurol using *n*-octane as an internal standard. **S10**

Building and testing the predictive model: **S11**

List of descriptors used *S11*

Calculating the model validation error *S13*

Ligand characterization:

Materials and Instrumentation

All reactions were carried out under atmospheric conditions, unless stated otherwise. Infrared (IR) spectra were obtained from neat samples using a Shimadzu FTIR-8400s spectrophotometerand with wavelengths (*v*) reported in cm^{-1}. ^{1}H and ^{13}C nuclear magnetic resonance (NMR) spectra were recorded on a Bruker Avance 250 (250.13 MHz for ^{1}H and 62.90 MHz for ^{13}C) or Bruker Avance 500 (500.23 MHz for ^{1}H and 125.70 MHz for ^{13}C) with chemical shifts (δ) reported in reported in ppm, internally referenced to residual solvent resonances for $CDCl_3$ (^{1}H δ: = 7.26 ppm; $^{13}C\{^{1}H\}$ δ: = 77.00 ppm), and coupling constants (*J*) are reported in Hz. Electrospray Ionisation (ESI) mass spectrometry was carried out with a micrOTOF-Q instrument in positive ion mode unless stated otherwise. Chromatographic purification refers to flash chromatography using the indicated solvent (mixture) and Baker 7024-02 silica gel (40 µ, 60 Å). Thin layer chromatography was performed using silica plates from Merck (Kieselgel 60 F254 on aluminium with fluorescence indicator. Compounds on TLC were visualised by UV detection unless stated otherwise. Dichloromethane (DCM) was dried and freshly distilled from CaH_2 prior to use. Other commercially available reagents were used as purchased.

General procedure I for the synthesis of 2*H*-2-imidazoline archetypes.

Seven archetypes of 2*H*-2-imidazolines (**A** - **G**) were synthesized via a three-component Mannich-type reaction. All reactions were performed using a concentration of 1 M of aldehyde, 1 M of amine, and 0.5 M of isocyanide in dry CH_2Cl_2 or MeOH. Na_2SO_4 and the aldehyde were added, at 25 ºC, to a stirred solution of the amine. The mixture was stirred for 2 h. The isocyanide was then added and the mixture was stirred at 25 ºC for an additional 18 h, filtered, and concentrated *in vacuo*. The crude product was purified by flash column chromatography (cyclohexane–EtOAc–Et_3N = 2:1:0.01, gradient, unless stated otherwise). All 2-*H*-2-midazolinium iodides [**A** – **D**][1], [**E** – **F**[2]] and **G**[3] were reported earlier.

[1] Strassberger, Z., Mooijman, M., Ruijter, E., Alberts, A. H., de Graaff, C., Orru, R. V. A., Rothenberg, G., *Appl. Organomet. Chem.* **2010**, *24*, 142.
[2] Bon, R. S., van Vliet, B., Sprenkels, N. E., Schmitz, R. F., de Kanter, F. J. J., Stevens, C. V., Swart, M., Bickelhaupt, F. M., Groen, M. B., Orru, R. V. A., *J. Org. Chem.* **2005**, *70*, 3542.
[3] Bon, R. S., Sprenkels, N. E., Koningstein, M. M., Schmitz, R. F., de Kanter, F. J. J., Domling, A., Groen, M. B., Orru, R. V. A., *Org. Biomol. Chem.* **2008**, *6*, 130.

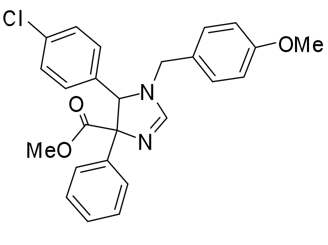

A

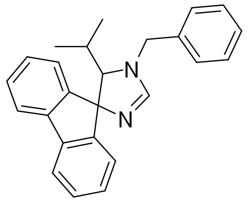

B

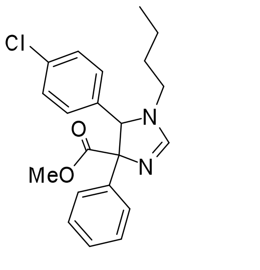

C

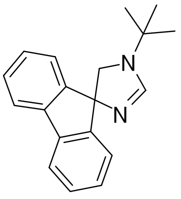

D

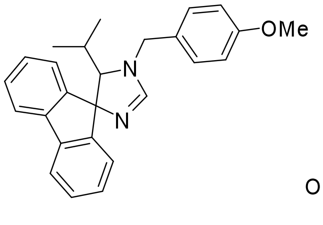

E

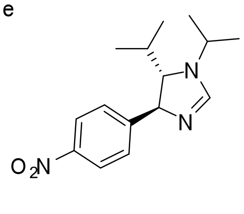

F

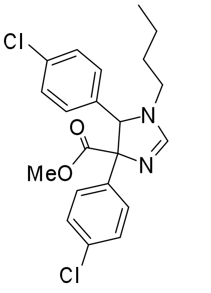

G

General procedure II for the synthesis of 2-imidazolinium salts.

Reactions were carried out at a concentration of 1 M of imidazoline in acetone. The alkyl halide (1 eq) was added to a stirred solution of the imidazoline and NaI (1 eq). The reaction mixture was stirred at rt for 18 h. Then, the reaction mixture was filtrated over Celite and concentrated *in vacuo*.

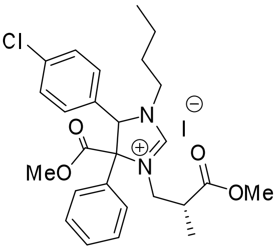

Imidazolinium iodide 7: According to general procedure II for the synthesis of 2-imidazolinium salts, the reaction between 2*H*-2-imidazoline **C** (100.0 mg, 0.268 mmol), NaI (40.0 mg, 0.268 mmol) and methyl (*S*)-3-bromo-2-methylpropanoate (51.0 mg, 36.2 µl, 0.268 mmol) afforded 2-imidazolinium iodide **7** (180.0 mg, 0.183 mmol, 69%) as a 5:2 mixture of diastereoisomers as a yellow foam. ^{1}H NMR (500 MHz, $CDCl_3$) δ 9.53 (s,1H), 9.47 (s, 1H), 7.69 (d, *J* = 7.5, 2H), 7.42 (d, *J* = 7.5, 2H), 7.40 – 7.31 (m, 4H), 7.30 – 7.27 (m, 2H), 7.01 – 6.99 (m, 9H), 5.91 (s, 1H), 5.28 (s, 1H), 3.87 – 3.84 (m, 2H) 3.67 (s, 3H), 3.63 (s, 3H), 3.49 (dd, *J* = 10.3, 6.7, 2H), 3.39 (dd, *J* = 10.3, 5.8, 2H), 3.28 (dd, *J* = 10.0, 6.5, 2H), 3.18 (dd, *J* = 10.3, 6.0, 2H), 3.08 – 3.02 (m,2H), 3.00 – 2.92 (m, 2H), 2.86 – 2.78 (m 1H), 2.76 – 2.68 (m, 1H), 3.15 (s, 1H), 2.077 (s, 1H), 1.58 – 1.53 (m, 2H), 1.45 – 1.38 (m, 2H), 1.26 – 1.17 (m, 4H), 1.20 (d, *J* = 7.0, 3H), 1.18 (d, *J* = 7.0, 2H), 0.81 (t, *J* = 7.5, 2H), 0.70 (t, *J* = 7.5, 2H); ^{13}C NMR (125 MHz, $CDCl_3$) δ 173.5 (C), 173.5 (C), 170.3 (C), 167.0 (C), 157.9 (CH), 156.1 (CH), 138.1 (C), 135.9 (C), 134.9 (C), 132.6 (C), 130.6 (C), 129.3 (C),

129.3 (CH), 129.1 (CH), 128.9 (CH), 128.5 (2x CH), 128.3 (2x CH), 125.9 (CH), 125.7 (2x CH), 125.2 (C), 78.2 (C), 76.8 (CH), 74.5 (CH), 53.8 (CH_3), 51.8 (CH_3), 46.4 (CH_2), 46.0 (CH_3), 41.8 (CH), 41.7 (CH), 29.2 (CH_2), 29.2 (CH_2), 19.3 (CH_2), 19.0 (CH_2), 18.0 (CH_3), 17.9 (CH_3), 13.3 (CH_3), 13.2 (CH_3); IR (neat): 3064, 2954, 2941, 2875, 1730, 1633, 1492, 1449, 1417, 1211, 1089, 1015, 734, 696, 488; HRMS calcd for $C_{26}H_{32}ClN_2O_4$ [M − I$^-$] 471.2045, found 471.2050.

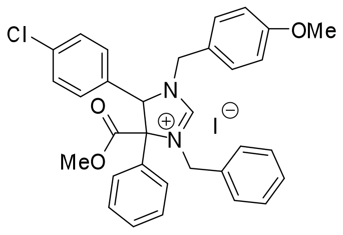

Imidazolinium iodide 8: According to general procedure II for the synthesis of 2-imidazolinium salts, the reaction between 2*H*-2-imidazoline **A** (100.0 mg, 0.230 mmol), NaI (40.0 mg, 0.27 mmol) and benzyl bromide (54.0 mg, 39.3 µl, 0.27 mmol) afforded 2-imidazolinium iodide **8** (150.1 mg, 0.229 mmol, 100%) as a 3:4 mixture of diastereoisomers as a yellow foam. ^{1}H NMR (500 MHz, $CDCl_3$) δ 10.09 (s, 1H), 9.99 (s, 1H), 7.44-7.31 (m, 16H), 7.25-7.19 (m, 3H), 7.11-7.01 (m, 3H), 7.01-6.97 (m, 3H), 6.88 (d, *J* = 8.5, 2H), 6.86 (d, *J* = 8.5, 2H), 5.912 (s, 1H), 5.48 (d, *J* = 14.0, 1H), 5.40 (d, *J* = 15.0, 1H), 5.72 (s, 1H), 5.17 (d, *J* = 15.0, 1H), 4.77 (d, *J* = 15.0, 1H), 4.62 (d, *J* = 14.0, 1H), 4.38 (d, *J* = 14.0, 1H), 4.21 (d, *J* = 15.0, 1H), 4.08 (d, *J* = 14.0, 1H), 3.81 (s, 6H), 3.61 (s, 3H), 3.26 (s, 3H); ^{13}C NMR (125 MHz, $CDCl_3$) δ 166.3 (C), 160.23 (C), 159.2 (CH), 157.3 (CH), 136.5 (C), 135.3 (C), 134.1 (C), 132.6 (C), 131.4 (CH), 131.2 (CH), 130.5 (2x CH), 130.4 (CH), 130.3 (2x CH), 130.3 (CH), 123.0 (C), 129.8 (CH), 129.6 (CH), 129.5 (2x CH), 129.4 (2x CH), 129.2 (2x CH), 129.2 (CH), 129.1 (CH), 129.0 (CH), 128.9 (CH), 128.9 (CH), 128.8 (2x CH), 128.7 (2x CH), 128.7 (CH), 128.6 (2x CH), 128.5 (C), 128.2 (CH), 127.7 (2x CH), 127.5 (CH), 127.4 (CH), 127.0 (CH), 123.3 (C), 122.9 (C), 114.5 (CH), 114.4 (CH), 81.2 (C), 80.9 (C), 73.9 (CH), 70.0 (CH), 55.3 (CH_3), 55.2 (CH_3), 53.9 (CH_3), 52.7 (CH_3), 51.2 (CH_2), 51.1 (CH_2), 50.6 (CH_2), 50.5 (CH_2), 50.4 (CH_2), 50.4 (CH_2); IR (neat): 3080, 3049, 2960, 2923, 2868, 1738, 1628, 1610, 1513, 1490, 1449, 1430, 1357, 1242, 1207, 1176, 1087, 1025, 824, 753, 695; HRMS calcd for $C_{26}H_{32}ClN_2O_4$ [M − I$^-$] 525.1939, found 525.1912.

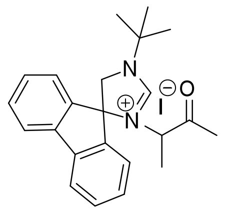

Imidazolinium iodide 14: According to general procedure II for the synthesis of 2-imidazolinium salts, the reaction between 2*H*-2-imidazoline **D** (100.0 mg, 0.362 mmol), NaI (54.0 mg, 0.360 mmol) and 3-chlorobutan-2-one (38.4 mg, 36.5 µl, 0.358 mmol) afforded 2-imidazolinium iodide **14** (119.5 mg, 0.252 mmol, 70 %) as an orange-brown oil. ^{1}H NMR

(250 MHz, $CDCl_3$) δ 9.12 (s, 1H), 7.71 – 6.78 (m, 3H), 7.54 – 7.45 (m, 3H), 7.33 – 7.32 (m, 2H), 4.39 (d, *J* = 11.9, 1H), 4.16 (d, *J* = 11.9, 1H), 3.53 – 3.44 (m, 1H), 1.80 (s, 3H), 1.742 (s, 9H), 1.63 (d, *J* = 7.2, 3H); ^{13}C NMR (63 MHz, $CDCl_3$) δ 203.5 (C), 156.4 (CH), 146.5 (C), 141.2 (C), 139.9 (C), 139.8 (C), 131.0 (CH), 130.9 (CH), 129.4 (CH), 128.6 (CH), 125.5 (CH), 124.3 (CH), 120.6 (CH), 120. 5 (CH), 74.8 (C), 59.1 (CH), 58.2 (CH_2), 56.1 (C), 27.7 (CH_3), 26.0 (CH_3), 18.1 (CH_3); IR (neat): 3069, 3029, 2947, 2918, 1700, 1617, 1369, 1304, 1232, 1173, 1092, 759, 733, 671, 640; HRMS calcd for $C_{23}H_{27}N_2O$ [M − I^-] 347.2118, found 347.2101.

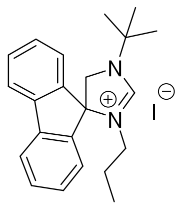

Imidazolinium iodide 15: According to general procedure II for the synthesis of 2-imidazolinium salts, the reaction between 2*H*-2-imidazoline **D** (100.0 mg, 0.362 mmol), NaI (54.0 mg, 0.360 mmol) and *n*-propyl bromide (44.3 mg, 32.8 µl, 0.360 mmol) afforded 2-imidazolinium iodide **14** (153.1 mg, 0.343 mmol, 95 %) as an orange-brown oil. ^1H NMR (500 MHz, $CDCl_3$) δ 9.65 (s, 1H), 7.73 – 7.69 (m, 4H), 7.51 (t, *J* = 7.2 , 2H), 7.47 (t, *J* = 7.2 , 2H), 4.223 (s, 2H), 3.14 (q, *J* = 7.2, 7.2, 2H), 1.68 (s, 9H), 1.33 - 1.25 (m, 2H), 0.69 (t, *J* = 7.4, 3H); ^{13}C NMR (125 MHz, $CDCl_3$) δ 155.9 (CH), 142.0 (C), 139.6 (C), 130.6 (CH), 128.9 (CH), 128.0 (CH), 124.3 (CH), 120.4 (CH), 74.4 (C), 57.5 (CH_2), 56.7 (CH_2), 46.7 (C), 27.7 (CH_3), 22.4 (CH_2), 10.5 (CH_3); IR (neat): 3075, 3023, 2968, 2876, 1701, 1622, 1575, 1447, 1370, 1232, 1178, 760, 734, 645; HRMS calcd for $C_{22}H_{27}N_2$ [M − I^-] 319.2169, found 319.2155.

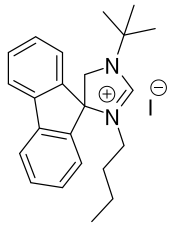

Imidazolinium iodide 16: According to general procedure II for the synthesis of 2-imidazolinium salts, the reaction between 2*H*-2-imidazoline **D** (100.0 mg, 0.362 mmol), NaI (54.0 mg, 0.360 mmol) and *n*-butyl bromide (49.3 mg, 38.8 µl, 0.360 mmol) afforded 2-imidazolinium iodide **16** (164.8 mg, 0.358 mmol, 100 %) as a light yellow foam. ^1H NMR (250 MHz, $CDCl_3$) δ 9.23 (s, 1H), 7.62 (d, *J* = 7.5, 2H), 7.53 (d, *J* = 7.5, 2H), 7.31 (t, *J* = 7.2, 2H), 7.22 (t, *J* = 7.2, 2H), 4.08 (s, 2H), 2.93 (t, *J* = 7.8, 2H), 1.50 (s, 9H), 1.10 - 1.06 (m, 2H), 0.88 – 0.84 (m, 2H), 0.42 (t, *J* = 7.0, 3H); ^{13}C NMR (63 MHz, $CDCl_3$) δ 155.2 (CH), 141.6 (C), 139.2 (C), 130.2 (CH), 128.5 (CH), 124.0 (CH), 120.0 (CH), 74.0 (C), 57.2 (C), 56.3 (CH_2), 44.5 (CH_2), 30.4 (CH_2), 27.3 (CH_3), 18.6 (CH_2), 12.5 (CH_3); IR (neat): 3075, 3023, 2975, 2865, 1622, 1450, 1375, 1300, 1298, 1212, 1200, 1184, 760, 733, 644; HRMS calcd for $C_{23}H_{29}N_2$ [M − I^-] 333.2325, found 333.2309.

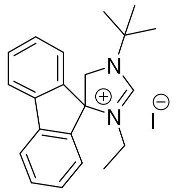

Imidazolinium iodide 17: According to general procedure II for the synthesis of 2-imidazolinium salts, the reaction between 2*H*-2-imidazoline **D** (100.0 mg, 0.362 mmol), NaI (54.0 mg, 0.360 mmol) and ethyl bromide (39.2 mg, 26.9 µl, 0.360 mmol) afforded 2-imidazolinium iodide **17** (155.6 mg, 0.359 mmol, 100%) as an orange foam. ^{1}H NMR (250 MHz, $CDCl_3$) δ 9.54 (s, 1H), 7.74 - 7.71 (m, 2H), 7.66 - 7.63 (m, 2H), 7.53 - 7.29 (m, 4H), 4.241 (s, 2H), 3.27 (q, J= 7.3, 2H), 1.68 (s, 9H), 0.99 (t, *J* = 7.3, 3H); ^{13}C NMR (63 MHz, $CDCl_3$) δ 155.8 (CH), 142.2 (C), 139.7 (C), 130.7 (CH), 129.0 (CH), 124.4 (CH), 120.4 (CH), 74.4 (C), 57.6 (C), 56.8 (CH_2), 40.4 (CH_2), 27.8 (CH_3), 14.9 (CH_3; IR (neat): 3075, 3023, 2968, 2935, 1701, 1622, 1576, 1450, 1373, 1298, 1238, 1186, 753, 732, 637; HRMS calcd for $C_{21}H_{25}N_2$ [M − I^-] 305.2012, found 305.1998.

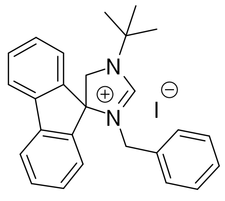

Imidazolinium iodide 18: According to general procedure II for the synthesis of 2-imidazolinium salts, the reaction between 2*H*-2-imidazoline **D** (100.0 mg, 0.362 mmol), NaI (54.0 mg, 0.360 mmol) and benzyl bromide (61.3 mg, 42.8 µl, 0.358 mmol) afforded 2-imidazolinium iodide **18** (134.2 mg, 0.271 mmol, 75 %) as an orange-brown foam. ^{1}H NMR (250 MHz, $CDCl_3$) δ 9.57 (s, 1H), 7.69 - 7.62 (m, 4H), 7.51 - 7.45 (m, 2H), 7.37 - 7.31 (m, 2H), 7.11 - 7.08 (m, 3H), 6.99 - 6.96 (m, 2H), 4.38 (s, 2H), 4.23 (s, 2H), 1.66 (s, 9H); ^{13}C NMR (63 MHz, $CDCl_3$) δ 155.9 (CH), 141.9(C), 140.0 (C), 133.3 (C), 130.7 (CH), 129.1 (CH), 129.0 (CH), 128.3 (CH), 128.2 (CH), 124.9 (CH), 120.4 (CH), 74.7 (C), 58.0 (CH_2), 57.2 (CH_2), 49.1 (C), 27.9 (CH_3); IR (neat): 3075, 3023, 2963, 2935, 2189, 1622, 1451, 1366, 1304, 1221, 917, 768, 721, 693; HRMS calcd for $C_{26}H_{27}N_2$ [M − I^-] 367.2169, found 376.2152.

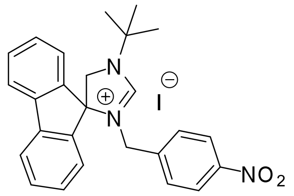

Imidazolinium iodide 19: According to general procedure II for the synthesis of 2-imidazolinium salts, the reaction between 2*H*-2-imidazoline **D** (100.0 mg, 0.362 mmol), NaI (54.0 mg, 0.360 mmol) and *p*-nitrobenzyl bromide (77.8 mg, 0.360 mmol) afforded 2-imidazolinium iodide **19** (194.1 mg, 0.359 mmol, 100 %) as a yellow foam. ^{1}H NMR (250 MHz, $CDCl_3$) δ 10.68 (s, 1H), 7.82 (d, *J* = 8.6, 2H), 7.80 (d, *J* = 7.2 Hz 2H), 7.48 (t, d, *J* = 7.5 Hz 2H), 7.38 - 7.26 (m, 4H), 7.11 (d, *J* = 8.6 Hz 2H), 4.70 (s, 2H), 4.253 (s, 2H), 1.68 (s, 9H); ^{13}C NMR (63 MHz, $CDCl_3$) δ 156.5 (CH), 146.7 (C), 141.2 (C), 140.0 (C), 139.4 (C), 130.1 (CH), 127.5 (CH), 124. 7 (CH), 122.3 (CH), 120.0 (CH), 74.1 C), 58.0 (CH_2), 56.7 (CH_2), 48.2 (C), 27.5 (CH_3); IR (neat): 3075, 3023, 2973, 2935, 1620, 1515, 1450, 1342, 1201, 1108,

1047, 946, 855, 769, 758, 734, 707, 643, 618; HRMS calcd for $C_{26}H_{26}N_3O_2$ [M − I⁻] 412.2020, found 412.2001.

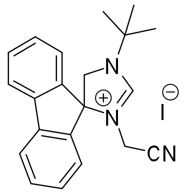

Imidazolinium iodide 20: According to general procedure II for the synthesis of 2-imidazolinium salts, the reaction between 2*H*-2-imidazoline **D** (100.0 mg, 0.362 mmol), NaI (54.0 mg, 0.360 mmol) and chloroacetonitrile (27.2 mg, 22.78 µl, 0.360 mmol) afforded 2-imidazolinium iodide **20** (159.6 mg, 0.360 mmol, 100 %) as a light yellow foam. ^{1}H NMR (500 MHz, $CDCl_3$) δ 9,99 (s, 1H), 7.84 (d, *J* = 7.5, 2H), 7.73 (d, *J* = 7.5, 2H), 7.58 - 7.54 (m, 2H), 7.49-7.45 (m, 2H), 4,55 (s, 2H), 1,71 (s, 2H), 4,32 (s, 9H); ^{13}C NMR (125 MHz, $CDCl_3$) δ 156.9 (CH), 140.4 (C), 140.2 (C), 131.9 (CH), 129.8 (CH), 125.3 (CH), 121.1 (CH), 112.6 (C), 74.8 (C), 59.3 (C), 57.3 (CH_2), 32.8 (CH_2), 27.7 (3x CH_3); IR (neat): 3011, 2992, 2883, 1636, 1448, 1313, 1267, 1109, 1060, 950, 901, 834, 768, 733; HRMS calcd for $C_{21}H_{22}N_3$ [M − I⁻] 316.1908 found 316.1795.

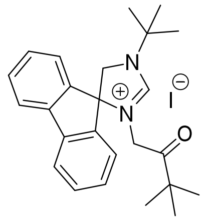

Imidazolinium iodide 21: According to general procedure II for the synthesis of 2-imidazolinium salts, the reaction between 2*H*-2-imidazoline **D** (100.0 mg, 0.362 mmol), NaI (54.0 mg, 0.360 mmol) and 1-bromo-4,4-dimethylpentan-3-one (64.1 mg, 47.3 µl, 0.358 mmol) afforded 2-imidazolinium iodide **21** (144.7 mg, 0.288 mmol, 80%) as an orange-brown foam. ^{1}H NMR (500 MHz, $CDCl_3$) δ 9.20 (s, 1H), 7.62 – 7.52 (m, 2H), 7.45 – 7.38 (m, 2H), 7.30 – 7.20 (m, 2H), 7.19 – 7.10 (m, 2H), 4.193 (s, 2H), 4.068 (s, 2H), 1.575 (s, 9H), 0.633 (s, 9H); ^{13}C NMR (125 MHz, $CDCl_3$) δ 207.3 (C), 157.7 (CH), 141.0 (C), 139.8 (C), 130.9 (CH), 128.8 (CH), 125.1 (CH), 120.2 (CH), 74.5 (C), 57.6 (C), 55.8 (CH_2), 47.8 (CH_2), 42.4 (C), 27.5 (CH_3), 24.9 (CH_3); IR (neat): 3023, 3011, 2982, 2970, 1718, 1617, 1451, 1369, 1307, 1214, 1053, 982, 941, 770, 734; HRMS calcd for $C_{25}H_{31}N_2O$ [M − I⁻] 375.2431 found 375.2417.

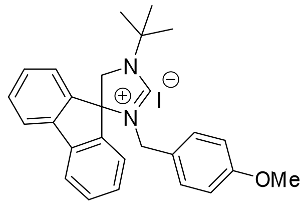

Imidazolinium iodide 22: According to general procedure II for the synthesis of 2-imidazolinium salts, the reaction between 2*H*-2-imidazoline **D** (100.0 mg, 0.362 mmol), NaI (54.0 mg, 0.360 mmol) and *p*-methoxybenzyl bromide (72.3 mg, 51.9 µl, 0.359 mmol) afforded 2-imidazolinium iodide **22** (188.8 mg, 0.360 mmol, 100 %) as a yellow foam. ^{1}H NMR (500 MHz, $CDCl_3$) δ 9,45 (s, 1H), 7,68 - 7,64 (m, 4H), 7,49 - 7,46 (m, 2H),

7,38 - 7,34 (m, 2H), 6.93 (d, $J = 2.0$, 2H), 6.58 (d, $J = 2.0$, 2H), 4.25 (d, $J = 38.0$, 2H), 3.75 (d, $J = 38.0$, 2H), 3.71 (s, 3H), 1,637 (2, 9H); ^{13}C NMR (125 MHz, $CDCl_3$) δ 159.5 (C), 155.5 (CH), 142.2 (C), 140.2 (C), 130.4 (CH), 129.9 (CH), 125.5 (C), 125.0 (CH), 120.6 (CH), 113.7 (CH), 57.3 (CH_2), 55.3 (CH_2), 55.3 (CH_3), 48.5 (CH_2), 31.0 (3x CH_3); IR (neat): 3005, 2977, 2958, 2842, 1610, 1506, 1451, 1300, 1245, 1174, 1108, 1028, 981, 734; HRMS calcd for $C_{27}H_{29}N_2O$ [M − I^-] 397.2274 found 397.2258.

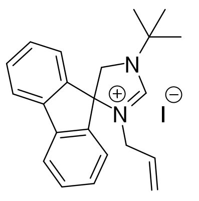

Imidazolinium iodide 24: According to general procedure II for the synthesis of 2-imidazolinium salts, the reaction between 2*H*-2-imidazoline **D** (297.2 mg, 1.07 mmol), NaI (160.5 mg, 1.07 mmol) and allyl chloride (81.9 mg, 87.7 µl, 1.07 mmol) afforded 2-imidazolinium iodide **24** (449.0 mg, 1.01 mmol, 94%) as a yellow foam. ^{1}H NMR (500 MHz, $CDCl_3$) δ 9.51 (s, 1H), 7.76 (d, $J = 7.2$, 2H), 7.68 (d, $J = 7.2$, 2H), 7.52 - 7.49 (m, 2H), 7.45 - 7.41 (m, 2H), 5.53 - 5.49 (m, 1H), 5.01 (dd, $J = 17.0, 0.8$, 1H), 4.91 (dd, $J = 10.0, 0.8$, 2H), 4.25 (s, 2H), 3.83 (d, $J = 7.0$, 2H), 1.68 (s, 9H); ^{13}C NMR (125 MHz, $CDCl_3$) δ 156.1 (CH), 142.3 (C), 140.2 (C), 131.1 (CH), 130.6 (CH), 129.3 (CH), 124.9 (CH), 121.5 (CH_2), 120.3 (CH), 74.6 (C), 58.1 (C), 57.2 (CH_2), 48.2 (CH_2), 28.0 (3x CH_3); IR (neat): 2982, 2959, 1617, 1452, 1375, 1302, 1272, 1207, 1047, 930, 733; HRMS calcd for $C_{22}H_{25}N_2$ [M − I^-] 317.2021 found 317.1996.

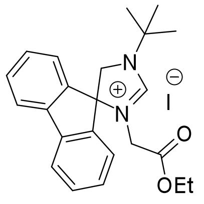

Imidazolinium iodide 25: According to general procedure II for the synthesis of 2-imidazolinium salts, the reaction between 2*H*-2-imidazoline **D** (100.0 mg, 0.362 mmol), NaI (54.0 mg, 0.360 mmol) and ethyl chloroacetate (44.1 mg, 31.1 µl, 0.359 mmol) afforded 2-imidazolinium iodide **25** (175.4 mg, 0.357 mmol, 99%) as a yellow foam. ^{1}H NMR (250 MHz, $CDCl_3$) δ 9.47 (s, 1H), 7.81 – 7.79 (d, $J = 7.2$, 2H) , 7.70 – 7.68 (d, $J = 7.5$, 2H), 7.53 – 7.38 (m, 4H), 4.27 (s, 2H), 4.21 (q, $J = 7.1$, 2H), 3.68 (s, 2H), 1.70 (s, 9H), 1.28 (t, $J = 7.1$, 3H); ^{13}C NMR (63 MHz, $CDCl_3$) δ 167.9 (C), 157.9 (CH), 141.5 (C), 140.3 (C), 131.2 (CH), 129.4 (CH), 125.6 (CH), 120.6 (CH), 75.0 (C), 62.4 (CH_2), 58.3 (CH_2), 56.9 (CH_2), 45.4 (C), 27.8 (CH_3), 13.9 (CH_3); IR (neat): 3075, 3023, 2979, 2935, 1726, 1701, 1622, 1451, 1448, 1375, 1309, 1265, 1194, 1902, 1027, 773, 734; HRMS calcd for $C_{23}H_{27}N_2O_2$ [M − I^-] 363.2067, found 363.2053.

Imidazolinium iodides [**4**, **9**, **13**][4], **6**[5] and [**10**, **11**, **12**, **26**][6] were synthesized as reported earlier. Imidazolium iodide **5** was purchased from commercial sources.

Procedures for performing control NMR experiments

NMR control experiment 1: In a flame-dried NMR tube and under inert atmosphere KO*t*Bu (11.2 mg, 99.8 μmol, 1eq) was added to a mixture of imidazolinium salt **26** (49.2 mg, 99.9 μmol, 1eq) and $[RuCl_2(p\text{-cymene})]_2$ (61.0 mg, 99.6 μmol, 1 eq) in THF (1 ml). The resulting reaction mixture was then stirred on a vortex mixer for 1 minute and subsequently ^1H and ^{13}C NMR measurements were performed. In the ^{13}C NMR analysis no Ru-C signal was observed. ^1H NMR and ^{13}C NMR measurements after 18h showed no change of the signals.

NMR control experiment 2: In a flame-dried NMR tube and under inert atmosphere KO*t*Bu (3.4 mg, 30.3 μmol, 1eq) was added to a solution of imidazolinium salt **26** (15.0 mg, 30.4 μmol, 1eq) in THF (1 ml). The resulting reaction mixture was stirred on a vortex mixer for 1 minute and subsequently ^1H and ^{13}C NMR measurements were performed. No dimerization product was observed. ^1H and ^{13}C NMR measurements after 18h showed no change of the signals.

NMR control experiment 3: In a flame-dried NMR tube and under inert atmosphere a 1M solution of KO*t*Bu (1eq) in THF was added dropwise over a period of 24h to a solution of imidazolinium salt **26** (15.0 mg, 30.4 μmol, 1 eq) in THF (1 ml). The resulting reaction mixture was stirred on a vortex mixer for 1 minute and subsequently ^1H and ^{13}C NMR measurements were performed. No dimerization product was observed. ^1H and ^{13}C NMR measurements after 18h showed no change of the signals.

---

[4] R. S. Bon, B. van Vliet, N .E. Sprenkels, R. F. Schmitz, F. J. J. de Kanter, C. V. Stevens, M. Swart, F. M. Bickelhaupt, M. B. Groen, R. V. A. Orru, *J. Org. Chem.* **2005**, *70*, 3542.

[5] R. S. Bon, N. E. Sprenkels, M. M. Koningstein, R. F. Schmitz, F. J. J. de Kanter, A. Dömling, M. B. Groen, R. V. A. Orru *Org. Biomol. Chem.* **2008**, *6*, 130.

[6] Z. Strassberger, M. Mooijman, E. Ruijter, A. H. Alberts, C. de Graaff, R. V. A. Orru, G. Rothenberg, *Appl. Organomet. Chem.* **2010**, *24*, 142.

GC calibration experiments for furfural and furfurol using *n*-octane as an internal standard.

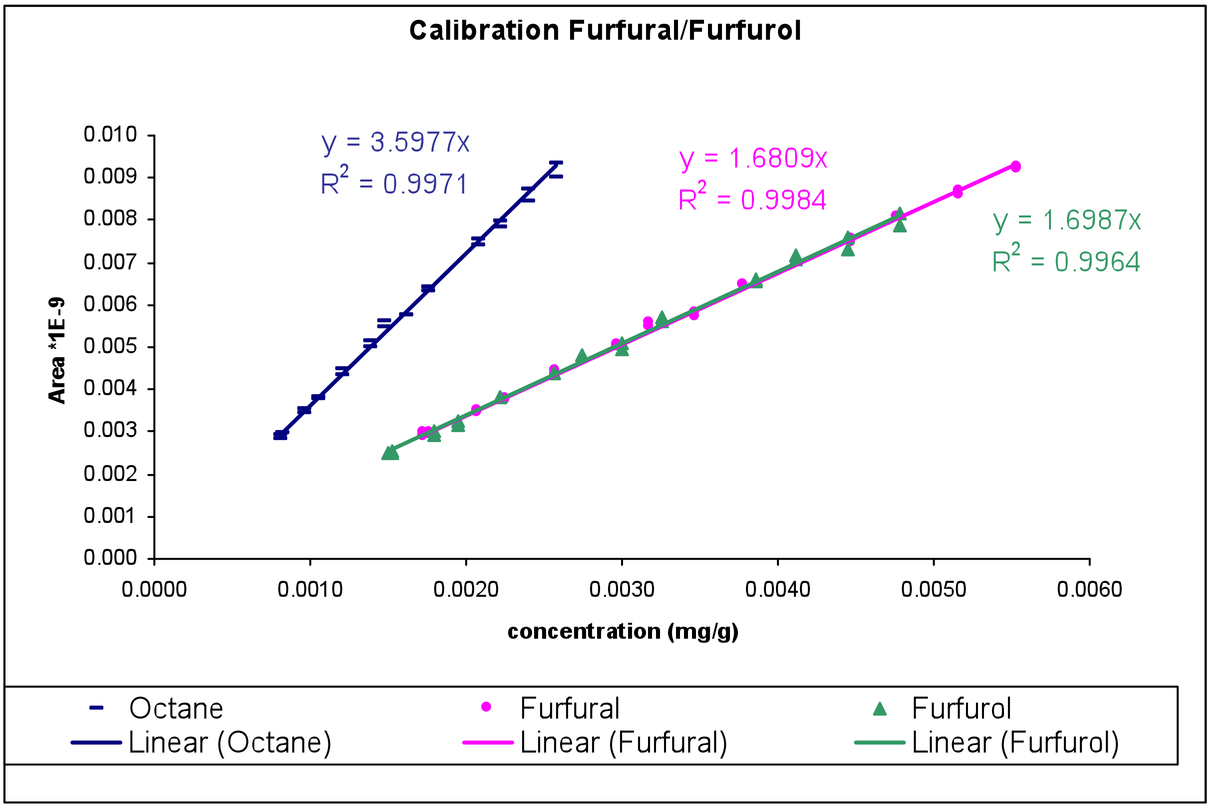

<u>Building and testing the predictive model:</u>

List of descriptors used

{1}=Number of atoms
{2}=Number of C atoms
{3}=Relative number of C atoms
{4}=Number of H atoms
{5}=Relative number of H atoms
{6}=Number of O atoms
{7}=Relative number of O atoms
{8}=Number of N atoms
{9}=Relative number of N atoms
{10}=Number of S atoms
{11}=Relative number of S atoms
{12}=Number of F atoms
{13}=Relative number of F atoms
{14}=Number of Cl atoms
{15}=Relative number of Cl atoms
{16}=Number of Br atoms
{17}=Relative number of Br atoms
{18}=Number of I atoms
{19}=Relative number of I atoms
{20}=Number of P atoms
{21}=Relative number of P atoms
{22}=Number of bonds
{23}=Number of single bonds
{24}=Relative number of single bonds
{25}=Number of double bonds
{26}=Relative number of double bonds
{27}=Number of triple bonds
{28}=Relative number of triple bonds
{29}=Number of aromatic bonds
{30}=Relative number of aromatic bonds
{31}=Number of rings
{32}=Relative number of rings
{33}=Number of benzene rings
{34}=Relative number of benzene rings
{35}=Molecular weight
{36}=Relative molecular weight
{37}=Gravitation index (all bonds)
{38}=Gravitation index (all pairs)
{39}=Wiener index
{40}=Randic index (order 0)
{41}=Randic index (order 1)
{42}=Randic index (order 2)
{43}=Randic index (order 3)
{44}=Kier&Hall index (order 0)
{45}=Kier&Hall index (order 1)
{46}=Kier&Hall index (order 2)
{47}=Kier&Hall index (order 3)
{48}=Kier shape index (order 1)
{49}=Kier shape index (order 2)
{51}=Kier shape index (order 3)
{52}=Kier flexibility index
{53}=Average Information content (order 0)
{54}=Information content (order 0)
{55}=Average Structural Information content (order 0)
{56}=Structural Information content (order 0)
{57}=Average Complementary Information content (order 0)
{58}=Complementary Information content (order 0)
{59}=Average Bonding Information content (order 0)
{60}=Bonding Information content (order 0)
{61}=Average Information content (order 1)
{62}=Information content (order 1)
{63}=Average Structural Information content (order 1)
{64}=Structural Information content (order 1)
{65}=Average Complementary Information content (order 1)
{66}=Complementary Information content (order 1)
{67}=Average Bonding Information content (order 1)
{68}=Bonding Information content (order 1)
{69}=Average Information content (order 2)
{70}=Information content (order 2)
{71}=Average Structural Information content (order 2)
{72}=Structural Information content (order 2)
{73}=Average Complementary Information content (order 2)
{74}=Complementary Information content (order 2)
{75}=Average Bonding Information content (order 2)
{76}=Bonding Information content (order 2)
{77}=Balaban index

{78}=Moment of inertia A
{79}=Moment of inertia B
{80}=Moment of inertia C
{81}=XY Shadow
{82}=XY Shadow / XY Rectangle
{83}=YZ Shadow
{84}=YZ Shadow / YZ Rectangle
{85}=ZX Shadow
{86}=ZX Shadow / ZX Rectangle
{87}=Molecular volume
{88}=Molecular volume / XYZ Box
{89}=Molecular surface area
{90}=Max partial charge for a C atom [Zefirovs PC]
{91}=Min partial charge for a C atom [Zefirovs PC]
{92}=Max partial charge for a H atom [Zefirovs PC]
{93}=Min partial charge for a H atom [Zefirovs PC]
{94}=Max partial charge for a O atom [Zefirovs PC]
{95}=Min partial charge for a O atom [Zefirovs PC]
{96}=Max partial charge for a N atom [Zefirovs PC]
{97}=Min partial charge for a N atom [Zefirovs PC]
{98}=Max partial charge for a F atom [Zefirovs PC]
{99}=Min partial charge for a F atom [Zefirovs PC]
{100}=Max partial charge for a Pd atom [Zefirovs PC]
{101}=Min partial charge for a Pd atom [Zefirovs PC]
{102}=Max partial charge (Qmax)
{103}=Min partial charge (Qmin)
{104}=Polarity parameter (Qmax-Qmin)
{105}=Polarity parameter / square distance
{106}=Topographic electronic index (all pairs) [Zefirovs PC]
{107}=opographic electronic index (all bonds) [Zefirovs PC]
{108}=TMSA Total molecular surface area [Zefirovs PC]
{109}=PPSA-1 Partial positive surface area [Zefirovs PC]
{110}=PNSA-1 Partial negative surface area [Zefirovs PC]
{111}=DPSA-1 Difference in CPSAs (PPSA1-PNSA1) [Zefirovs PC]
{112}=FPSA-1 Fractional PPSA (PPSA-1/TMSA) [Zefirovs PC]
{113}=FNSA-1 Fractional PNSA (PNSA-1/TMSA) [Zefirovs PC]
{114}=WPSA-1 Weighted PPSA (PPSA1*TMSA/1000) [Zefirovs PC]
{115}=WNSA-1 Weighted PNSA (PNSA1*TMSA/1000) [Zefirovs PC]
{116}=PPSA-2 Total charge weighted PPSA [Zefirovs PC]
{117}=PNSA-2 Total charge weighted PNSA [Zefirovs PC]
{118}=DPSA-2 Difference in CPSAs (PPSA2-PNSA2) [Zefirovs PC]
{119}=FPSA-2 Fractional PPSA (PPSA-2/TMSA) [Zefirovs PC]
{120}=FNSA-2 Fractional PNSA (PNSA-2/TMSA) [Zefirovs PC]
{121}=WPSA-2 Weighted PPSA (PPSA2*TMSA/1000) [Zefirovs PC]
{122}=WNSA-2 Weighted PNSA (PNSA2*TMSA/1000) [Zefirovs PC]
{123}=PPSA-3 Atomic charge weighted PPSA [Zefirovs PC]
{124}=PNSA-3 Atomic charge weighted PNSA [Zefirovs PC]
{125}=DPSA-3 Difference in CPSAs (PPSA3-PNSA3) [Zefirovs PC]
{126}=FPSA-3 Fractional PPSA (PPSA-3/TMSA) [Zefirovs PC]
{127}=FNSA-3 Fractional PNSA (PNSA-3/TMSA) [Zefirovs PC]
{128}=WPSA-3 Weighted PPSA (PPSA3*TMSA/1000) [Zefirovs PC]
{129}=WNSA-3 Weighted PNSA (PNSA3*TMSA/1000) [Zefirovs PC]
{130}=RPCG Relative positive charge (QMPOS/QTPLUS) [Zefirovs PC]
{131}=RPCS Relative positive charged SA (SAMPOS*RPCG) [Zefirovs PC]
{132}=RNCG Relative negative charge (QMNEG/QTMINUS) [Zefirovs PC]
{133}=RNCS Relative negative charged SA (SAMNEG*RNCG) [Zefirovs PC]
{134}=HDSA H-donors surface area [Zefirovs PC]
{135}=FHDSA Fractional HDSA (HDSA/TMSA) [Zefirovs PC]

{136}=HASA H-acceptors surface area [Zefirovs PC]
{137}=FHASA Fractional HASA (HASA/TMSA) [Zefirovs PC]
{138}=HBSA H-bonding surface area [Zefirovs PC]
{139}=FHBSA Fractional HBSA (HBSA/TMSA) [Zefirovs PC]
{140}=HDCA H-donors charged surface area [Zefirovs PC]
{141}=FHDCA Fractional HDCA (HDCA/TMSA) [Zefirovs PC]
{142}=HACA H-acceptors charged surface area [Zefirovs PC]
{143}=FHACA Fractional HACA (HACA/TMSA) [Zefirovs PC]
{144}=HBCA H-bonding charged surface area [Zefirovs PC]
{145}=FHBCA Fractional HBSA (HBSA/TMSA) [Zefirovs PC]
{146}=min(#HA, #HD) [Zefirovs PC]
{147}=count of H-acceptor sites [Zefirovs PC]
{148}=count of H-donors sites [Zefirovs PC]
{149}=HA dependent HDSA-1 [Zefirovs PC]
{150}=HA dependent HDSA-1/TMSA [Zefirovs PC]
{151}=HA dependent HDSA-2 [Zefirovs PC]
{152}=HA dependent HDSA-2/TMSA [Zefirovs PC]
{153}=HA dependent HDSA-2/SQRT(TMSA) [Zefirovs PC]
{154}=HA dependent HDCA-1 [Zefirovs PC]
{155}=HA dependent HDCA-1/TMSA [Zefirovs PC]
{156}=HA dependent HDCA-2 [Zefirovs PC]
{157}=HA dependent HDCA-2/TMSA [Zefirovs PC]
{158}=HA dependent HDCA-2/SQRT(TMSA) [Zefirovs PC]
{159}=HASA-1 [Zefirovs PC]
{160}=HASA-1/TMSA [Zefirovs PC]
{161}=HASA-2 [Zefirovs PC]
{162}=HASA-2/TMSA [Zefirovs PC]
{163}=HASA-2/SQRT(TMSA) [Zefirovs PC]
{164}=HACA-1 [Zefirovs PC]
{165}=HACA-1/TMSA [Zefirovs PC]
{166}=HACA-2 [Zefirovs PC]
{167}=HACA-2/TMSA [Zefirovs PC]
{168}=HACA-2/SQRT(TMSA) [Zefirovs PC]

Calculating the model validation error

To compute the error and the ΔFOM model validation, we have used the following expressions:

$$\Delta FOM = | expFOM - predFOM |$$

$$\%error = | \Delta FOM / expFOM | \times 100$$

The experimental FOM (expFOM) is the reference value. The predicted FOM (predFOM) is the value to be validated.

Comparing the predicted FOM with the experimental results, we observe an average prediction error of around 3%. This is a good prediction power taking in account that several validation iterations are commonly necessary to achieve such a low percentage of error. The tendency of the prediction is also correct, giving high predicted values for experimentally measured high FOMs, and slightly lower predicted values for the experimentally measured low FOMs. We should point out that all the ligands used for building and testing the model

are properly explained by the PLS model. The unstructured residual small left plot in Figure S2, confirms this tendency.

Figure S1: Root mean square error of calibration, versus the 12 computed latent variables (LV) of the PLS model

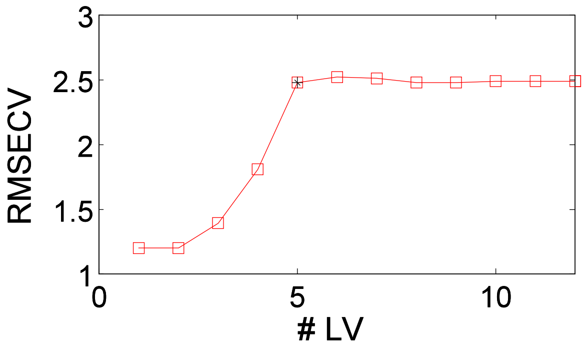

Figure S2: Residuals plot

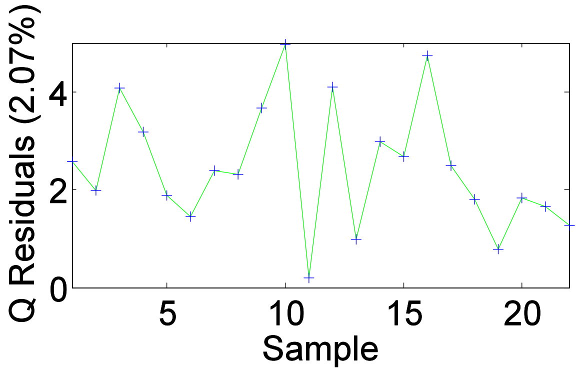

Supplement: Supplementary file 1 [file adsc0352-2201-SD1.pdf]
